# Artificial intelligence for early endometrial cancer diagnosis using multimodal clinical data: integrating deep learning, explainability, and data privacy

**DOI:** 10.3389/frai.2026.1787508

**Published:** 2026-04-13

**Authors:** Sital Dash, Kailas Patil, Arjun Bali, Ishwari Rohit Raskar, Yashwant Dongre, Amol Bhosle, Vishal Meshram

**Affiliations:** 1 Department of Computer Engineering, Vishwakarma University, Pune, Maharashtra, India; 2 Department of Business Analytics, University of Texas at Dallas, Dallas, TX, United States; 3 Department of Computer Science and Engineering, School of Computing, MIT Art, Design and Technology University, Pune, Maharashtra, India; 4 Vishwakarma Institute of Technology, Pune, Maharashtra, India

**Keywords:** clinical decision support, deep learning, endometrial cancer, explainable AI, histopathology whole-slide imaging, medical data fusion, multimodal artificial intelligence, privacy-aware learning

## Abstract

**Introduction:**

Early diagnosis of endometrial cancer is critical for improving survival. However, most AI-based methods are developed using single-modality data, and most of them are uninterpretable and do not offer privacy protection, or they completely ignore privacy issues. We propose a multimodal AI framework that utilizes histopathology whole-slide images (WSIs) and clinical data and incorporates both explainability and privacy-aware learning.

**Methods:**

Five hundred and twenty-nine patients’ data (354 early-stage and 175 advanced-stage) with 794 WSIs and 208,000 image patches were collected for the study. By using a convolutional neural network (CNN), morphological features were extracted from WSIs, and clinical variables were encoded by a multi-layer perceptron, respectively. These two modalities of information (the learned representations) were combined to make the final category prediction. Interpretability was enabled through Grad-CAM and clinical feature attribution, and privacy-aware training was supported by secure parameter aggregation.

**Results:**

The multimodal model obtained an accuracy of 0.91 and an AUC of 0.95, thus it exceeded the performance of clinical-only (accuracy = 0.78, AUC = 0.81) and histopathology-only (accuracy = 0.85, AUC = 0.89) models with a considerable increase in sensitivity (0.89) and specificity (0.93). The performance was preserved by privacy-aware learning as well. The net clinical benefit was maximum as per the Decision Curve Analysis.

**Conclusion:**

This framework offers a solution that is accurate, interpretable, and privacy-preserving, thus it can act as a diagnostic aid for early endometrial cancer. However, since the model was developed and evaluated using only the TCGA-UCEC cohort, external multi-center validation is required to confirm generalizability across diverse clinical populations, imaging protocols, and laboratory conditions.

## 1 Introduction

The global rise in gynecological cancers places a substantial burden on healthcare systems, with one of the main issues being the difficulty of identifying cases early and the lack of fair access to timely care (Echle et al., 2021). Endometrial cancer is one of the most affected types of cancers that has received close attention because it is gradually becoming more common and clinical outcomes heavily depend on early diagnosis (Echle et al., 2020). Patients with endometrial cancer at the early stage usually have good chances of survival, but if the diagnosis is delayed, the patient will most likely develop an advanced stage disease, treatment will be more complicated, and the overall prognosis will be worse (Kather et al., 2019). Considering this, deep learning (DL) has attracted a lot of interest as an effective tool for increasing the accuracy of diagnoses and thus, facilitating clinical decision-making by means of recognizing patterns in complicated and varied medical data (Wagner et al., 2023). Nevertheless, the deployment of AI-based diagnostic tools in everyday clinical settings is still limited by issues of the lack of transparency of the methods used, concerns about data confidentiality, and difficulties in embedding them in the clinical workflow (Hong et al., 2021).

### 1.1 Background and clinical challenges in endometrial cancer diagnosis

Most endometrial cancer cases are diagnosed by surgically removing tissue samples followed by histopathological investigation of biopsy. These are often supported by imaging techniques like transvaginal ultrasound and magnetic resonance imaging (MRI) (National Cancer Institute, n.d.). These methods are effective for clinical purposes; however, they require a lot of resources, are dependent on the operator, and might not be able to detect subtle pathological changes of the early-stage consistently (Guo et al., 2021). Different levels of diagnostic accuracy between various hospitals and healthcare professionals make early detection more difficult, especially in the settings where resources are scarce (Campanella et al., 2019). Due to these constraints, there is a demand for widely available, non-invasive, and data-driven diagnostic support systems that can help doctors to make clinical decisions on the identification of high-risk individuals even at the initial stage of the disease.

### 1.2 Artificial intelligence in cancer diagnosis

Artificial intelligence has made huge leaps recently, especially through deep learning, and these developments have profound implications for medical diagnosis. In particular, they allow for an automatic feature extraction from complex data such as histopathology images, radiological scans, and electronic health records (EHRs) (Chen et al., 2023; Hou et al., 2026). Deep learning techniques in cancer have been exploited for detecting tumors, classifying, grading, and predicting prognoses which, in many cases, are at par or outperform traditional methods (Huang et al., 2021). In the treatment of endometrial cancer, AI techniques aid in the analysis of histopathological images, accurate imaging-based staging, and risk stratification through clinical variables (Cheerla and Gevaert, 2019). However, a majority of the articles still focus on single-modality data, thereby restricting their potential to replicate actual clinical decision-making scenarios.

### 1.3 Importance of multimodal learning

Clinical diagnosis in oncology is inherently multimodal as it integrates data from histopathology, medical imaging, and patient’s clinical information. Multimodal deep learning is a method that seeks to merge such diverse data sources in order to provide a single, more comprehensive representation of disease characteristics (Holzinger et al., 2019). Various research in different cancer areas have indicated that multimodal methods can bring about better diagnostic reliability, less uncertainty, and higher model translatability compared to uni-modal models (Tjoa and Guan, 2021). However, only a handful of multimodal AI approaches have been created so far for early endometrial cancer diagnosis, and their clinical utility has not been thoroughly examined.

### 1.4 Explainability in AI-based medical diagnosis

Deep learning models, however, are typically criticized for the black-box nature that limits transparency and clinical trust despite their good predictive performance (Lipton, 2018). Explainability is very important in healthcare, where clinicians explain their decisions and only then rely on AI-generated predictions for diagnosis and treatment (Guidotti et al., 2018). Explainable artificial intelligence (XAI) techniques such as saliency mapping, attention mechanisms, and feature attribution methods are intended to increase model interpretability and align AI outputs with clinical reasoning (Topol, 2019). Consequently, including explainability is highly important for gaining clinician acceptance and preventing the careless use of AI in medical diagnosis.

### 1.5 Data privacy and ethical considerations

Along with the massive usage of large-scale medical datasets, the patient privacy, data security, and regulatory compliance issues have become the biggest concern (Kaissis et al., 2020). Medical data of person are very sensitive, and if they are revealed or used without consent, the resulting consequences can both be ethically and legally quite severe. In order to solve these problems and at the same time enable local and global model development, privacy-aware learning paradigms have been proposed, which also include decentralized and controlled data-sharing methods (McMahan et al., 2017). However, the emphasis on data privacy is usually diminished in AI-based diagnostic studies, which lowers their readiness level for the real-world deployment.

### 1.6 Research gap

While there is a rising trend of AI utilization for diagnosing endometrial cancer, the existing research still exhibits a number of substantial gaps:

- G1: A majority of the present works concentrate on single-modality data only, thus they are less representative of clinical scenario and less robust for diagnosis.
- G2: Most of the time, explainability has either been missing or has been inadequate, which limits how much clinicians can understand and rely on the system.
- G3: Medical data is highly sensitive, but hardly any studies have considered data privacy when developing their models.
- G4: At the moment, no single framework exists that can concurrently deal with early diagnosis, multimodal learning, explainability, and data privacy aspects in endometrial cancer.

### 1.7 Research questions

To address these gaps, this study is guided by the following research questions:

RQ1: Can multimodal deep learning models integrating histopathology, imaging, and clinical data improve early endometrial cancer diagnostic performance compared to single-modality approaches?

RQ2: How can explainable AI techniques be effectively incorporated to enhance transparency and clinical interpretability of endometrial cancer predictions?

RQ3: What privacy-aware learning strategies can be employed to protect sensitive patient data without significantly compromising diagnostic accuracy?

RQ4: How does the proposed integrated framework perform in terms of robustness, clinical relevance, and potential for real-world deployment?

### 1.8 Study contributions

This study answers the raised research questions by suggesting an AI-powered solution that uses multimodal deep learning, interpretability tools, and privacy safeguards for early endometrial cancer identification. In doing so, this research makes a significant step forward in the creation of healthcare- and ethics-friendly AI systems that are clinically reliable and capable of helping women’s health and public health decisions beyond the present work.

## 2 Related work

### 2.1 AI-based diagnostic approaches for endometrial cancer

One of the first uses of AI in endometrial cancer was deep learning of histopathological whole-slide images, whereby malignant tissue and relevant morphological patterns were reliably identified (Rieke et al., 2020). After that, researchers have been able to show the cancer detection and grading at the level of clinical-grade performance, hence allowing for the routine diagnostic workflow (Sheller et al., 2020). Co-application of computational pathology and biomarker discovery has resulted in even better tumor characterization based on AI (Papernot et al., 2017).

Besides that, AI models aided by imaging techniques such as ultrasound and magnetic resonance imaging have been investigated as means of detecting tumors, staging and assessing the extent of myometrial invasion (Dwork and Roth, 2014). However, these purely imaging-based methods, although they have shown potential, have not solved the problem completely, especially when used separately, and thus, pathological or clinical information have been overlooked (Li et al., 2020).

### 2.2 Multimodal deep learning in oncology (RQ1)

Multimodal deep learning in oncology has become a focus of research to overcome the limitations of unimodal methods. Overviews indicate that diagnostics become more robust and predictive performance gets better when heterogeneous data sources are integrated (Talhouk et al., 2015). The digitization of pathology and pan-cancer analyses are shown to be strong examples where the combination of tissue-level features with molecular or clinical data provides even greater insight (Subramanian et al., 2020).

Deep learning being used in healthcare generally always points to multimodal fusion being crucial to achieving clinically meaningful outcomes. It has been suggested that the combination of artificial intelligence and clinician knowledge can lead to the best medical decisions, especially in diagnostic cases that are intricate and complex. Very detailed and clear reporting methods such as TRIPOD-AI encourage the use of multimodal prediction models that can be easily understood and duplicated (Sala et al., 2023; Litjens et al., 2017; Madabhushi and Lee, 2016).

### 2.3 Explainable artificial intelligence for clinical decision support

Explainability has become a major focus of AI integration in medicine as the models have become more complex. One way to interpret deep learning predictions is through the use of visualization-based methods such as gradient-weighted class activation mapping which identifies the diagnostically relevant areas (Kather et al., 2020). Outline of features of explainable AI frameworks include a set of standards for transparency and accountability in healthcare applications (Levine and Levine, 2013).

Explainable AI methods are widely recognized in a number of surveys as a way of enabling clinicians to have more trust in what the AI is saying and to make decisions based on an informed understanding of the AI (Talhouk et al., 2016). The use of model-agnostic explanation methods to further increase the level of interpretability of state-of-the-art complex classifiers for both medical imaging and clinical prediction tasks is indeed a trend in recent research (Bibault et al., 2016).

### 2.4 Data privacy and federated learning in healthcare AI

The increasing use of large-scale medical datasets for analysis has raised concerns about patient privacy and data governance. However, privacy-preserving machine learning methods show that it is possible to train deep learning models without the sharing of centralized data (Gevaert et al., 2017). Federated learning is a technique that has been identified as a feasible way to develop models cooperatively and yet keep the data confidential (Huang et al., 2017).

Studies involving multiple institutions have been done to verify the use of federated learning in medical imaging and they have emphasized the scalability and applicability of the method for healthcare AI (Samek et al., 2017). Furthermore, the broader scope of digital health further emphasizes that privacy-aware methods are definitely a must for the safe deployment of AI (Gunning and Aha, 2019).

### 2.5 Clinical translation, ethics, and governance

Among the necessities for clinical translation of AI systems are ethical principles, regulatory standards, and strict validation. Analyses of medical big data stress transparency, accountability, and a patient-centered design as the main characteristics (Char et al., 2018). Several international guidelines provide policy-level proposals for the responsible use of AI in healthcare (Sheller et al., 2023). A comprehensive ground truth of AI vs. clinician performance has delineated the potentials and shortcomings of AI in medicine at this time (Shokri and Shmatikov, 2015). It has been suggested that any deep learning application for medical image analysis must be subjected to rigorous external validation and bias assessment before clinical translation (Selvaraju et al., 2020).

### 2.6 Advanced applications and emerging directions

Recently, researchers have combined proteogenomic and molecular data with AI models to improve diagnostic and risk stratification abilities (Barredo Arrieta et al., 2023). By using large endometrial cancer cohorts, prognostic modeling helps to move AI applications beyond simply diagnosis (Sheller et al., 2023). Molecular sub type analyses are yet another way by which personalized risk assessment is supported (Liu et al., 2023).

Biomarker-driven diagnostic modeling shows how integrated data approaches can be valuable for clinical decision support (Collins et al., 2020). New multimodal deep learning pipelines provide methods that can be used not only for one cancer type but also for others (Arvaniti et al., 2019). A review of precision oncology thus confirmed the clinical importance of integrated AI systems (Roberts et al., 2021).

Explainable AI frameworks are still evolving and their focus is more on clinical usability (Ariyaratne et al., 2023). Massive-scale surveys are well-organized and showcase the best practices for federated learning in the field of healthcare analytic (Riley et al., 2021). A thorough analysis of explainable AI methods brings together the most important factors that will enable the responsible and clinically meaningful use of AI (Zhang et al., 2022).

### 2.7 Synthesis of related work

Existing research has confirmed that the use of artificial intelligence in the diagnosis of endometrial cancer using histopathology, imaging, and clinical data is very effective; however, most of the methods are still restricted to single data modalities and very specific diagnostic tasks. Though multimodal deep learning, explainable artificial intelligence, and privacy-preserving learning have each frankly demonstrated upside when done separately, the combination of these methods in a single diagnostic system for early detection of endometrial cancer has been hardly touched. This disintegration is indicative of a big misalignment between the technical breakthroughs and the clinic-ready solutions which serves as a good reason for building integrated, transparent, and privacy-aware AI systems that are well suited to real-world diagnostic workflows.

A concise methodological comparison with representative state-of-the-art studies is presented in Table 1 which summarizes representative recent methods for endometrial-cancer classification from histopathology that are relevant for comparison (Hong et al., 2021; Zhang et al., 2022; Frémond et al., 2023). Note that many published works evaluate on different datasets and under different experimental protocols; the table is therefore intended for methodological comparison rather than direct numeric benchmarking.

**Table 1:** Comparison with representative state-of-the-art methods for endometrial cancer histopathology analysis.

Study	Year	Data modality	Dataset	Aggregation strategy	Explainability	Privacy-aware	Key contribution
Hong et al. (2021) (Panoptes)	2021	Multi-resolution WSIs	TCGA	Multi-resolution CNN with patch aggregation	Limited	No	Predicts molecular subtypes from histology using multi-scale inputs
Zhang et al. (2022)	2022	WSIs (H&E)	Institutional dataset	CNN with patch aggregation	No	No	Automated endometrial cancer detection on clinical slides
Frémond et al. (2023)	2023	WSIs (H&E)	Institutional cohort	Interpretable deep learning	Yes	No	Interpretable molecular prediction validated with pathology insight
Proposed Study	2026	WSIs + Clinical data (multimodal)	TCGA-UCEC	Mean pooling + multimodal fusion	Yes (Grad-CAM + feature attribution)	Yes (simulated)	Integrates multimodal learning, explainability, and privacy-aware training

## 3 Methodology

To improve clarity and reproducibility, the methodology is organized into five structured components: (i) problem formulation and data preprocessing, (ii) modality-specific feature learning, (iii) multimodal fusion and prediction, (iv) explainability and privacy-aware learning, and (v) algorithmic and statistical evaluation. Mathematical formulations are presented alongside their corresponding modules, while Algorithm 1 provides a consolidated workflow summary. Section 3.1–3.3 now focus on problem formulation and preprocessing. Section 3.4–3.6 present model architecture and mathematical formulation. Section 3.8 presents privacy-aware aggregation equations. Section 3.9 (Algorithm 1) provides a concise workflow summary. Section 3.10 isolates statistical analysis.

**Figure 1:**
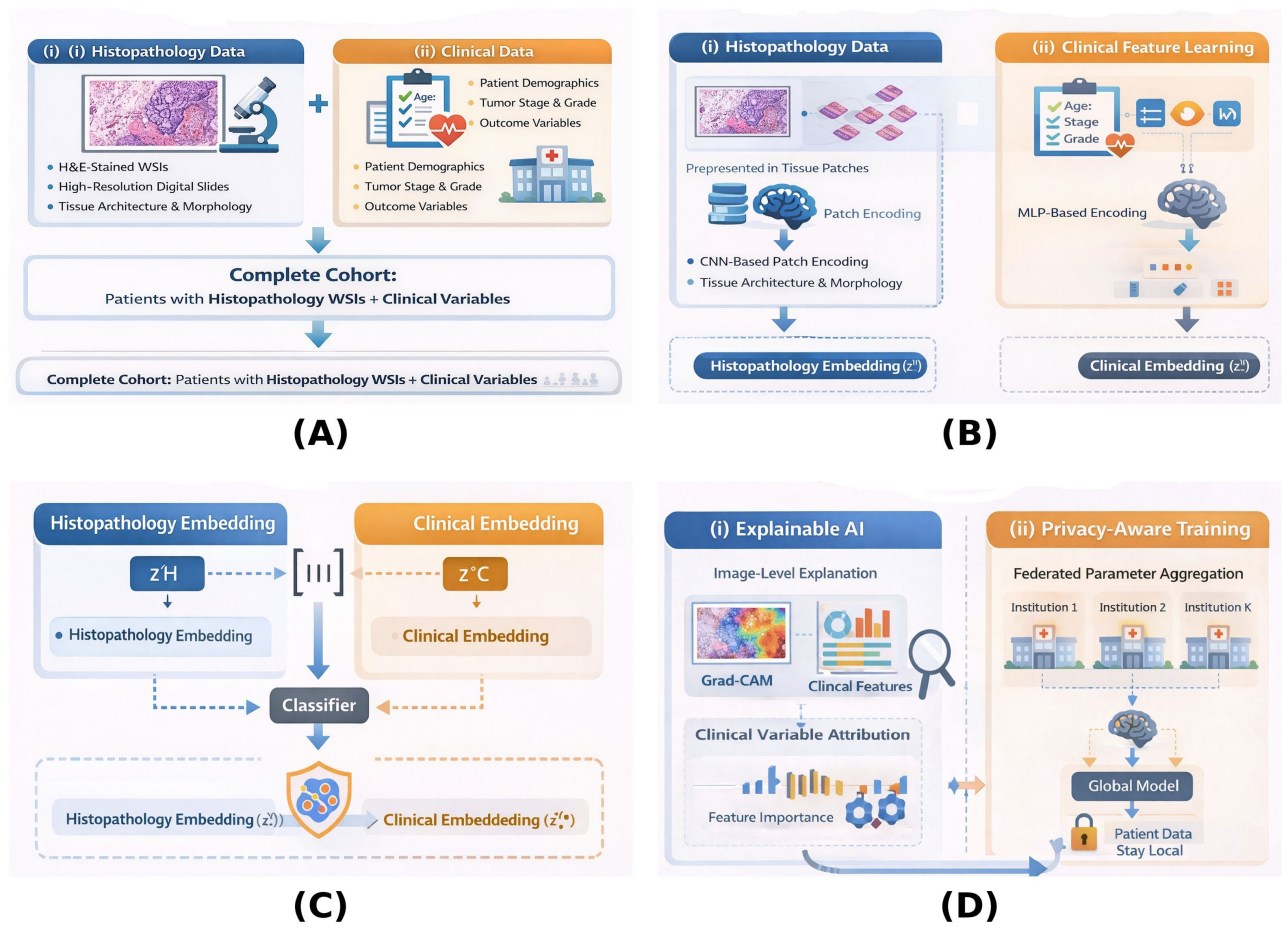
Overview of the proposed multimodal AI framework for early endometrial cancer diagnosis. **(A)** Multimodal data inputs. **(B)** Modality-specific feature learning. **(C)** Multimodal fusion and diagnostic prediction. **(D)** Explainability and privacy-aware learning.

This study proposes a multimodal artificial intelligence framework for early endometrial cancer diagnosis that integrates histopathology whole-slide images (WSIs) and structured clinical data. The framework is designed in four stages as shown in Figure 1: (A) Data acquisition, (B) Modality-specific feature learning, (C) Multi modal fusion and prediction, and (D) Explainability and privacy-aware learning. The overall objective is to achieve accurate, interpretable, and privacy-preserving diagnosis suitable for clinical and public-health deployment.

### 3.1 Study design and problem formulation

A retrospective design is adopted using publicly available, deidentified datasets. The diagnostic task is formulated as a binary classification problem shown in Equation 1.


$$F:(H_{i},C_{i})\to y_{i}$$ (1)

where, $H_{i}$ is the histopathology WSI of patient i, $C_{i}$ is the structured clinical feature vector, and $y_{i}$ ∈{0,1} denotes early-stage (0) or advanced-stage (1) endometrial cancer.

### 3.2 Datasets description, source, and data availability

As illustrated in Figure 1A, the proposed framework uses two complementary input modalities: histopathology whole-slide images (H&E-stained) and structured clinical data, which together provide both tissue-level and patient-level information for diagnosis.

#### 3.2.1 Data source

The primary dataset used is the TCGA-UCEC (Uterine Corpus Endometrial Carcinoma) cohort from The Cancer Genome Atlas. TCGA-UCEC is a large-scale, well-curated cancer dataset widely used in computational pathology and clinical AI research.

The dataset contains three complementary components:

1 Histopathology Whole-Slide Images (WSIs):

- H&E-stained diagnostic slides digitized at high resolution.
- Each WSI typically contains billions of pixels, capturing tissue architecture and cellular morphology.
- These images serve as the primary source for morphological feature extraction.

2 Clinical Data:

- Demographic variables: age at diagnosis.
- Tumor characteristics: FIGO stage, histological grade, histological subtype.
- Outcome variables: survival time and status (used for analysis but not directly as prediction targets).

3 Molecular and Genomic Annotations

- Molecular subtypes and genomic mutation profiles.
- Used for stratification and validation, not for training the main classifier.

Only patients satisfying both of the following were included: availability of at least one high-quality WSI, and availability of complete essential clinical metadata. This ensures strict multimodal consistency.

#### 3.2.2 Data access links

- Clinical and metadata: https://portal.gdc.cancer.gov
- Histopathology WSIs: https://www.cancerimagingarchive.net/collection/tcga-ucec/

#### 3.2.3 Dataset size and cohort statistics

After applying inclusion criteria (availability of both histopathology WSIs and complete clinical metadata), Table 2 shows the final cohort size. This table includes the number of patients, distribution of early and advanced stage endometrial cancer cases, total number of histopathology whole-slide images (WSIs), and the scale of extracted image patches. The figures emphasize the various modes of data in the dataset and quantitatively justify it as a training and validation dataset for the proposed deep learning framework.

**Table 2:** Summary of the dataset composition and cohort statistics used in this study.

Data component	Count
Total patients included	529
Patients with valid WSIs	529
Patients with complete clinical data	529
Early-stage endometrial cancer cases (Stage I–II)	354
Advanced-stage endometrial cancer cases (Stage III–IV)	175
Total number of whole-slide images (WSIs)	794
Average WSIs per patient	≈1.50
Total extracted image patches	2,08,000
Average patches per WSI	≈262

The final cohort size of 529 patients was determined by strict inclusion criteria requiring the simultaneous availability of high-quality WSIs and complete essential clinical metadata. TCGA-UCEC contains additional cases with partial data; however, these were excluded to ensure multimodal consistency and avoid missing-modality bias. Thus, the cohort represents the maximum number of patients meeting all multimodal requirements rather than an arbitrarily selected sample size.

The dataset was split at the patient level into training (70%), validation (15%), and test (15%) sets using stratified sampling to preserve class distributions and prevent data leakage. Because stratified patient-level splitting was used, the class distribution in the test set closely mirrored the full cohort, with approximately a 2:1 early-to-advanced stage ratio. Therefore, the prevalence of advanced-stage cancer in the test set was approximately 33%. To mitigate potential optimism of AUC under class imbalance, we additionally report sensitivity, specificity, precision, F1-score, and decision-curve analysis.

### 3.3 Data preprocessing

#### 3.3.1 Histopathology preprocessing

**Figure 2:**
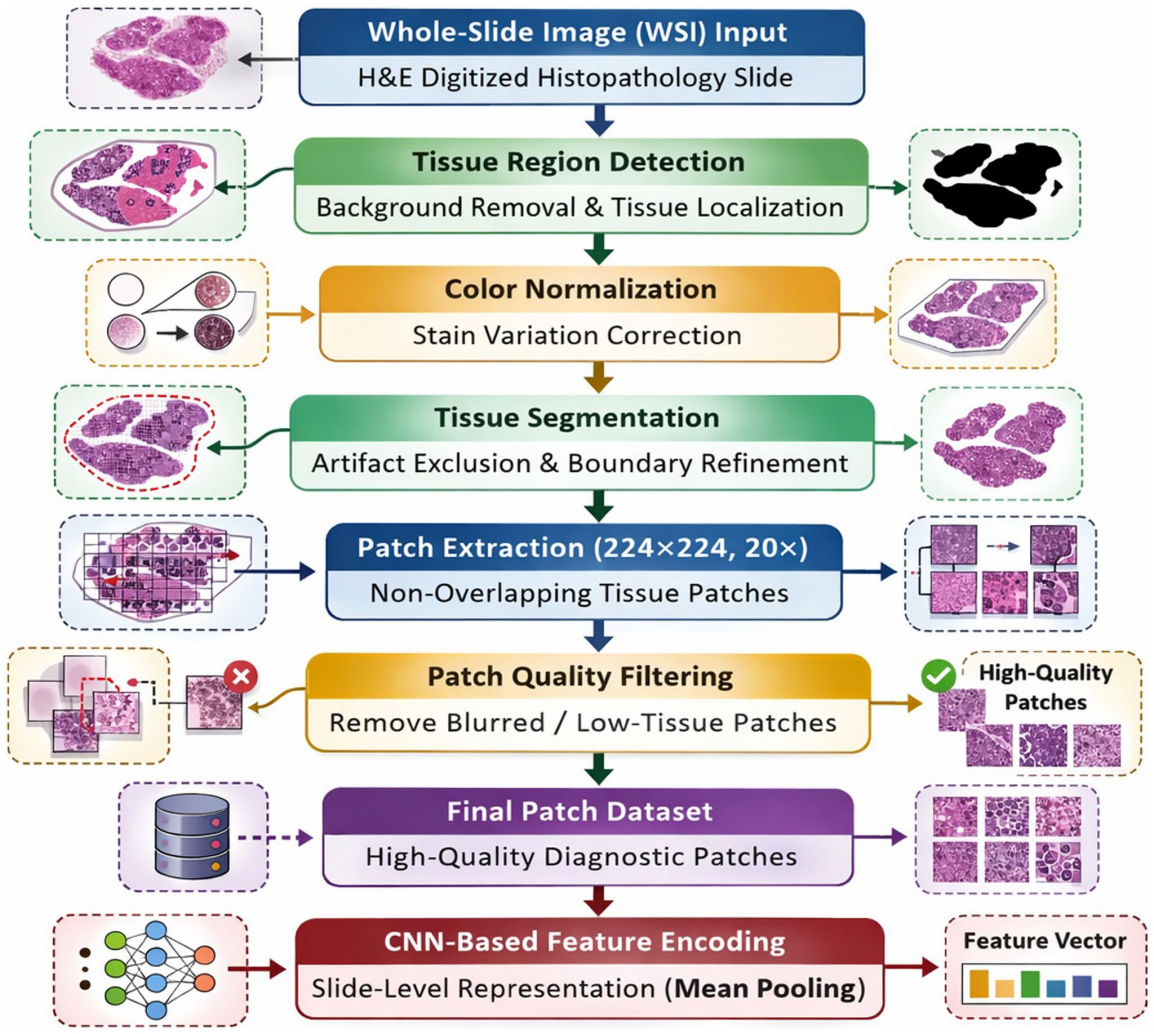
Standardized preprocessing and feature encoding workflow for histopathology whole-slide images (WSIs). The pipeline includes tissue detection, stain normalization, segmentation, patch extraction, quality filtering, and CNN-based feature encoding to generate slide-level representations for multimodal learning.

Histopathology whole-slide images (WSIs) are very large and have a lot of empty space, variations of staining, and imaging defects. These factors alone can worsen the performance of a deep learning model if they live directly in the training data. Hence, a well-thought-out preprocessing pipeline is crucial to convert raw WSIs into uniform and high-quality inputs that visually correspond to tissue morphology. Whole-slide images were divided into non-overlapping patches extracted at 20 × magnification with a patch size of 224 × 224 pixels, which is consistent with standard practice in computational pathology. In essence, the preprocessing step aims at denoising, normalizing visual features, and segmenting sections rich in tissue in a manner that a convolutional neural network can effectively utilize these features. WSI undergoes a series of systematic stages like the ones on the flowchart Figure 2 to attain the extracted image patches’ consistency, robustness, and clinical relevance. This stepwise instruction thus presents the described preprocessing workflow step-by-step.

##### 3.3.1.1 Whole-slide image (WSI) input

First the process involves acquiring high-resolution H&E-stained whole-slide images from biopsy or surgical tissue specimens for diagnostics. The images consist of both tissue and large background areas.

##### 3.3.1.2 Tissue region detection

The areas that do not have tissue or contain artifacts are removed by detecting only the tissue-containing regions. Hence, the step of unnecessary computation is eliminated and the analysis is concentrated on the biologically meaningful parts of the image only.

##### 3.3.1.3 Color normalization

The differences that arise from different staining protocols, scanners, and laboratory environments are reduced by adjusting the color distribution of WSIs to the same standard. In this way, the performance of the deep learning model across different images is enhanced and atmospheric variables do not affect the results.

##### 3.3.1.4 Tissue segmentation

The segmentation of the normalized picture helps to determine the exact boundaries of the valid tissue regions. In order to avoid the interference of noisy inputs, non-tissue components like folds, bubbles, or debris can be excluded.

##### 3.3.1.5 Patch extraction

The tissue parts obtained through segmentation are cut into square image patches (e.g., 256 × 256 pixels) at a certain level of magnification implemented throughout the whole tissue area. This allows CNNs to effectively process one large WSI split into considerably smaller pieces of the memory and computation scale.

##### 3.3.1.6 Patch quality filtering

Extracted patches are screened to remove: Patches with low tissue content, Blurred or out-of-focus regions, Patches dominated by background. This step guarantees that only the patches which contain sufficient information and thereby are diagnostically relevant are incorporated.

##### 3.3.1.7 Final patch dataset generation

The final patch dataset is obtained by gathering all the remaining high-quality patches together. The dataset is then fed as input into a convolutional neural network to learn histopathological features.

##### 3.3.1.8 CNN-based feature encoding

Subsequent to producing a high-quality patch dataset, each patch of tissue is sent through a convolutional neural network (CNN) encoder for hierarchical feature extraction. The CNN acquires discriminative morphological representations that include glandular architecture, nuclear atypia, stromal organization, and tumor microenvironment features that are pertinent to the diagnosis of endometrial cancer. Each patch-wise feature embeddings are then combined to get a slide-level representation which is used in the downstream multimodal fusion framework. The exact mathematical formulation and architectural configuration of the histopathology encoder are given in Section 3.5.1.

This step-wise approach effectively transforms the raw, large-scale histopathology slides into a standardized, clean, and information-rich dataset, which is then utilized for an accurate and robust deep learning based multimodal framework analysis.

#### 3.3.2 Clinical data preprocessing

Clinical data are complex and obtained from various sources; such datasets may contain continuous, ordinal, and categorical variables besides having some missing or inconsistent records. To make such data fit for deep learning, an elaborated preprocessing pipeline is applied which transforms the raw clinical information into a clean, standardized, and numerically stable feature representation. This step is very helpful in eliminating scale differences bias, handling incomplete records, and at the same time preserving the clinical relevance of patient attributes.

Normalization of continuous variables: in the first place, all continuous clinical variables (such as age and measurement values derived from the laboratory, if available) are subjected to standardized methods in which the mean is first subtracted and then the result divided by the standard deviation method using z-score normalization as explained in Equation 2.


$$x^{,}=\frac{x-\mu }{\sigma }$$ (2)

where x is the original value, μ denotes the mean of the variable, and σ is the standard deviation of the variable calculated from the training data. This standardization ensures that each continuous feature has an equal influence on the model training and that variables with large numerical ranges do not overshadow the learning of other variables.

##### 3.3.2.1 Categorical encoding

One-hot encoding is used for categorical variables like tumor stage, histological grade, and histological sub type, to convert them into numerical format. A binary vector represents each category, so the model can understand non-ordinal, non-linear relationships between different clinical classes without artificially ordering them. Thus, this transformation maintains the categorical data as separate entities but makes them neural network compatible.

##### 3.3.2.2 Missing data handling

Missing values are treated in several ways; in general, to avoid bias and leakage of data, strategies based on clinical knowledge were used.

- If the percentage of missing entries in a variable is less than 5%, such missing values are filled by the median of the variable (if continuous) or the mode (if categorical). This method is in line with the statistical aspects of the data and causes the least variation of the original distribution.
- A variable which has more than 5% missing values, in this case, will be left out of the analysis to avoid the risk of the model learning wrong features and being biased.

These limiting values (thresholds) are a compromise between the amount and the quality of data.

Feature vector construction: after the processes of normalization, encoding, and imputation, the clinical variables, which had been individually processed, were joined together as a whole to form the final numerical feature vector associated with each patient as shown in Equation 3.


$$C_{i}=[\;C_{i1},C_{i2},\ldots ,C_{\mathrm{im}}]$$ (3)

where, $C_{i}\;$ denotes the clinical feature vector of patient i, and m denotes the total number of clinical features processed. This vector is the normalized input of the clinical encoder network and facilitates seamless integration with histopathology features extracted in the multimodal learning framework.

On the whole, such a preprocessing pipeline guarantees that clinical data are strong, expandable, and in complete agreement with deep learning, yet their clinical interpretability and diagnostic significance are preserved.

### 3.4 Model architecture and training details

The detailed architecture and training configuration of the histopathology encoder are provided in Table 3.

**Table 3:** Implementation details of the proposed framework.

Component	Specification
Histopathology encoder	ResNet-50 CNN pretrained on ImageNet
Fine-tuning strategy	Last two residual blocks unfrozen for training
Input patch size	224 × 224 pixels
Patch aggregation	Mean pooling across patches
Clinical feature model	Multilayer Perceptron (MLP)
MLP layers	3 fully connected layers
Neurons per layer	256 → 128 → 64
Activation function	ReLU
Dropout	0.5
Fusion strategy	Late fusion (concatenation of embeddings)
Optimizer	Adam
Learning rate	1 × 10^−4^
Batch size	32
Epochs	50
LR scheduling	ReduceLROnPlateau
Early stopping	Based on validation loss
Data split strategy	Patient-level split
Training set	70%
Validation set	15%
Test set	15%
Split type	Stratified by class
Framework	PyTorch
Hardware	NVIDIA GPU

During training, data augmentation techniques including random horizontal and vertical flips, rotations, random cropping, and color jittering were applied to improve generalization and reduce overfitting.

### 3.5 Modality-specific feature learning

#### 3.5.1 Histopathology encoder

As illustrated in Figure 1B, histopathology whole-slide images are processed through a modality-specific feature learning pipeline in which each slide is decomposed into smaller tissue patches, encoded using a convolutional neural network, and aggregated to generate a slide-level representation. For patient i, the WSI is decomposed as shown in Equation 4.


$$H_{i}=\{\;p_{i1},p_{i2},\ldots ,p_{\text{in}}\}$$ (4)

where each $p_{\mathrm{ij}}\;$ corresponds to a fixed-size tissue patch (e.g., 256 × 256 pixels) extracted from biologically relevant regions of the slide after preprocessing.

Each patch is independently passed through a convolutional neural network (CNN) encoder $f_{\theta }\;(\cdot )$ , parameterized by θ, which learns hierarchical visual features from low-level textures to high-level tissue morphology as shown in Equation 5.


$$z_{\mathrm{ij}}=f_{\theta }(p_{\mathrm{ij}})$$ (5)

Here, $z_{\mathrm{ij}}$ denotes the latent feature representation of the j^th^ patch from patient i. The CNN captures diagnostically meaningful patterns such as glandular structures, nuclear atypia, stromal changes, and tumor micro environment characteristics that are crucial for endometrial cancer classification.

Since diagnostic decisions are made at the slide or patient level rather than the patch level, the patch-wise embedding must be aggregated to form a single holistic representation of the WSI. This is achieved through an aggregation function which is shown in Equation 6 implemented in this study using mean pooling.


$$z_{i}^{H}=\frac{1}{x}\;\sum_{j=1}^{n}z_{\mathrm{ij}}$$ (6)

where $z_{i}^{H}$ represents the slide-level histopathology embedding for patient i. This embedding captures the overall morphological information present in all tissue patches of the WSI and gives a concise but comprehensive representation of the patient’s histopathological features.

Mean pooling was selected for its simplicity, computational efficiency, and stability when training on moderately sized datasets. Unlike attention-based aggregation, mean pooling introduces no additional trainable parameters, thereby reducing the risk of overfitting when the number of patients is limited. Prior studies in computational pathology have also shown that mean pooling can provide competitive performance when patch sampling sufficiently captures tissue variability. Therefore, it was adopted as a robust baseline aggregation strategy.

The resulting histopathology embedding $z_{i}^{H}$ serves as the modality-specific feature vector that encodes tumor morphology, tissue organization, and cellular-level abnormalities. This representation forms one of the two core inputs to the multimodal fusion stage, where it is combined with the clinical feature embedding to enable integrated, patient-level diagnostic prediction.

#### 3.5.2 Clinical encoder

As illustrated in Figure 1B, the clinical encoder is designed to transform structured patient-level variables into a compact and informative feature representation that can be effectively integrated with histopathology-derived features. Clinical variables include demographic information and tumor-specific characteristics such as age, FIGO stage, histological grade, and histological subtype. These variables contain complementary diagnostic and prognostic information that reflects patient risk profiles and disease progression patterns.

Let $C_{i}$ denotes the processed clinical feature vector for patient i, obtained after normalization, encoding, and missing value handling. The clinical encoder is implemented as a Multi Layer Perceptron (MLP) $g_{\phi }\;(\cdot )$ , parameterized by ϕ, which consists of a sequence of fully connected layers with nonlinear activation functions as shown in Equation 7.


$$z_{i}^{C}=g_{\phi }(C_{i})$$ (7)

where $z_{i}^{C}\;$ represents the learned clinical embedding for patient I.

The MLP architecture enables the model to learn complex and nonlinear relationships among clinical variables that are difficult to capture using linear models. For example, the interaction between patient age and tumor stage, or between histological grade and subtype, may have a non-additive influence on disease severity and diagnostic outcomes. By applying nonlinear transformations, the MLP can capture such dependencies and encode them into a low-dimensional latent space.

Typically, the clinical encoder comprises: an input layer matching the dimensionality of the clinical feature vector, one or more hidden layers with nonlinear activation functions (e.g., ReLU), optional dropout layers for regularization, and an output layer that produces the clinical embedding.

This embedding is meant to be small, continuous, and distinctive, expressing the entire clinical profile of the patient in a way that is directly comparable and compatible with the histopathology embedding $z_{i}^{H}$ .

The generated clinical embedding $z_{i}^{C}$ effectively represents a variety of patient-specific elements such as risk factors, tumor characteristics, and the patterns of disease severity. It is, thus, the second major component of the multimodal representation. The next fusion stage (Section 3.5) will see this embedding being combined with the histopathology embedding to allow co-inference of tissue-level morphology and patient-level clinical context, thus increasing the reliability and clinical benefit of the diagnostic outputs.

### 3.6 Multimodal fusion and prediction

The multimodal fusion stage is the central decision-making part of the proposed framework, where the data from histopathology and clinical data are combined to make the final diagnostic prediction, as shown in Figure 1C. This stage aims to integrate different modality representations that complement each other and were learnt separately into a common feature space that reflects both the morphological features of the tissue and the clinical context of the patient.

Let $z_{i}^{H}$ be the histopathology embedding extracted from the CNN-based encoder (Section 3.4.1) and $z_{i}^{C}$ be the clinical embedding extracted from the MLP-based encoder (Section 3.4.2) for patient i. The two embeddings are merged by concatenating the features as shown in Equation 8.


$$z_{i}=[z_{i}^{H}\|z_{i}^{C}]$$ (8)

where || denotes the concatenation operator. This operation keeps the whole information content of both modalities intact by simply placing them next to each other in a higher-dimensional feature vector. The fused representation $z_{i}$ serves as a comprehensive multimodal descriptor of the patient, encoding morphological, pathological, and clinical attributes simultaneously.

Next, a fully connected classification layer takes the fused embedding for the final prediction as shown in Equation 9.


$${\hat{y}}_{i}=\sigma (Wz_{i}+b)$$ (9)

Where, W is the weight matrix of the classifier, b is the bias term, σ(.) denotes the sigmoid activation function, and ${\hat{y}}_{i}$ ∈ [0,1] represents the predicted probability that patient i belongs to the advanced-stage endometrial cancer class.

A probability threshold (typically 0.5) is applied to convert ${\hat{y}}_{i}$ into a binary diagnostic decision, distinguishing between early-stage and advanced-stage disease.

To train the model, the binary cross-entropy loss function is minimized and the loss function is shown in Equation 10.


$$L=-\frac{1}{N}\;\sum_{i=1}^{N}[y_{i}\;\log({\hat{y}}_{i})+(1-y_{i})\;\log(\;1-{\hat{y}}_{i}\;)]$$ (10)

where, N is the total number of training samples, $y_{i}$ ∈ {0,1} is the ground-truth label for patient i, and ${\hat{y}}_{i}$ is the predicted probability.

Because the dataset exhibits moderate class imbalance (354 early-stage vs. 175 advanced-stage cases, approximately 2:1), class-weighted binary cross-entropy loss was applied. Higher weight was assigned to the advanced-stage class to reduce bias toward the majority class and ensure balanced learning.

This loss function penalized the model for its incorrect guesses by imposing a higher loss on the confident but wrong predictions, thus push the model to output correct and well-calibrated probability estimates.

The fusion approach by concatenation was picked due to its ease, steadiness, and efficiency in multimodal learning. It gives each modality the ability to keep its independent representational power and at the same time the classifier during the training is able to learn cross-modal interactions. By optimization of the classifier layer parameters, the model is able to understand how morphological patterns from histopathology together with patient-level clinical risk factors determine diagnostic outcomes.

Medically, using this fusion technique gives the model the freedom to make its judgments not just by looking at the microscopic features of the tissue, such as the glandular architecture and nuclear atypia, but also by considering the macroscopic clinical factors, like tumor stage and histological grade. The modality integration step illustrated in Figure 1C transforms the single modality embedding into one unified diagnostic representation which is the basis for precise and reliable prediction of early endometrial cancer.

### 3.7 Explainable artificial intelligence

To further clarify Figure 1D, the scheme includes explainable artificial intelligence (XAI) elements to promote the transparency of the framework as well as to facilitate the clinical interpretability of model predictions. Given that medical AI systems should be accurate and also able to give reasoning that is understandable and verifiable by clinicians, the post-hoc explainability feature becomes a crucial part of the method suggested.

#### 3.7.1 Image explainability

We produce image-level explainability for histopathology data by applying Gradient-weighted Class Activation Mapping (Grad-CAM). Grad-CAM produces heatmaps (colored regions) that tell which areas (patches) of an image influenced a model’s prediction the most. The highlighted areas frequently correspond to tissue structures that are diagnostically relevant and recognizable, e.g., glandular morphology, nuclear atypia, stromal patterns, tumor infiltration.

Given a CNN map $A^{k}$ and the prediction score y, Grad-CAM computes the importance weights as shown in Equation 11.


$$\alpha_{k}=\frac{1}{Z}\;\sum_{i}\sum_{j}\frac{\partial y}{\partial A_{\mathrm{ij}}^{k}}$$ (11)

where, $A_{\mathrm{ij}}^{k}$ denotes the activation at location (i,j) of feature map k, and Z is a normalization factor.

The final Grad-CAM heatmap $L_{\text{Grad}-\mathrm{CAM}}$ is obtained as shown in Equation 12.


$$L_{\text{Grad}-\mathrm{CAM}}=\text{ReLU}\;(\sum_{k}\alpha_{k}A^{k})$$ (12)

The heatmap is combined with the original histopathology image to visually depict the tissue areas that led to the model’s decision. As illustrated in Figure 1D, this gave pathologists an opportunity to evaluate if the model concentrated on biologically relevant areas only and not on the background or artifacts.

#### 3.7.2 Clinical explainability

Clinical data explainability is done by feature attribution methods that indicate how much each clinical variable contributed to the final prediction. These methods give each feature in the clinical embedding an importance score showing the extent to which it influenced the diagnostic result.

For a clinical feature vector $C_{i}$ , the importance of each variable $c_{\mathrm{ij}}$ is determined from its effect on the prediction probability ${\hat{y}}_{i}$ . Variables such as tumor stage, histological grade, or patient age, if given high attribution scores, mean that the model is using clinically sensible risk factors.

As can be seen in Figure 1D, clinical feature attribution offers a clear understanding of patient-level decision drivers so that clinicians can check if the predictions are consistent with medical knowledge. Moreover, the image-level and clinical explainability results jointly constitute a comprehensive post-hoc interpretability framework. They change a deep learning model from a “black box” into a transparent decision-support system that clinicians can inspect, validate, and trust.

### 3.8 Privacy-aware learning

The privacy-aware learning setup in this study is a simulation of federated learning, rather than a deployment across physically separate institutions. Specifically, the TCGA-UCEC dataset was partitioned into K subsets to emulate data residing at different institutions. This approach allows controlled experimentation but does not fully reproduce real-world federated environments where data originate from independent sources.

In our simulation, K = 5 institutions were emulated. The dataset was partitioned into approximately equal-sized, stratified subsets to preserve class balance across institutions. The proposed framework also features a privacy-aware learning strategy, which is demonstrated in Figure 1D, to reflect real scenarios of multi-institutional deployment where data sharing between patient records is prohibited locally due to legal, ethical, and regulatory reasons.

Rather than pooling the original data, the framework adopts a federated-style learning approach. The dataset is split into K parts, each corresponding to a separate institution. A local model is trained separately on each subset, and only the learned model parameters are shared with a central aggregation server.

The global model parameters are evaluated as per Equation 13.


$$\theta^{\text{global}}=\frac{1}{K}\;\sum_{k=1}^{K}\theta_{k}$$ (13)

where, $\theta_{k}$ denotes the parameters of the model trained at institution k, K is the number of participating institutions, and $\theta^{\text{global}}$ is the aggregated global model.

Crucially, no raw patient data, histopathology images, or clinical records are exchanged between institutions. Only model parameters or gradients are communicated, significantly reducing the risk of data leakage and preserving patient confidentiality. This privacy-aware strategy provides several advantages:

- Data confidentiality: Sensitive medical data remain within their original institutions.
- Regulatory compliance: The framework aligns with healthcare data protection regulations.
- Scalability: Multiple hospitals can collaboratively train a stronger model without data sharing.
- Practical deployment: The approach reflects realistic clinical data governance structures.

The core of the idea shown in Figure 1D is that privacy-aware learning and explainability are two sides of one coin, which together make the ethical readiness of the framework stronger. On the one hand, explainability contributes to earning clinical trust through transparency, whereas on the other, privacy-aware learning guarantees safe and responsible use of data. Hence, coupling these aspects, the suggested multimodal AI system is not only clinically interpretable but also ethically ready for being deployed in the actual healthcare environment. As a result, the present arrangement is very similar to federated learning operation, but should be considered a simulation study instead of a real multi-institutional federated deployment.

### 3.9 Algorithmic summary

The entire training and inference workflow of the proposed framework is outlined in Algorithm 1.

**ALGORITHM 1:**
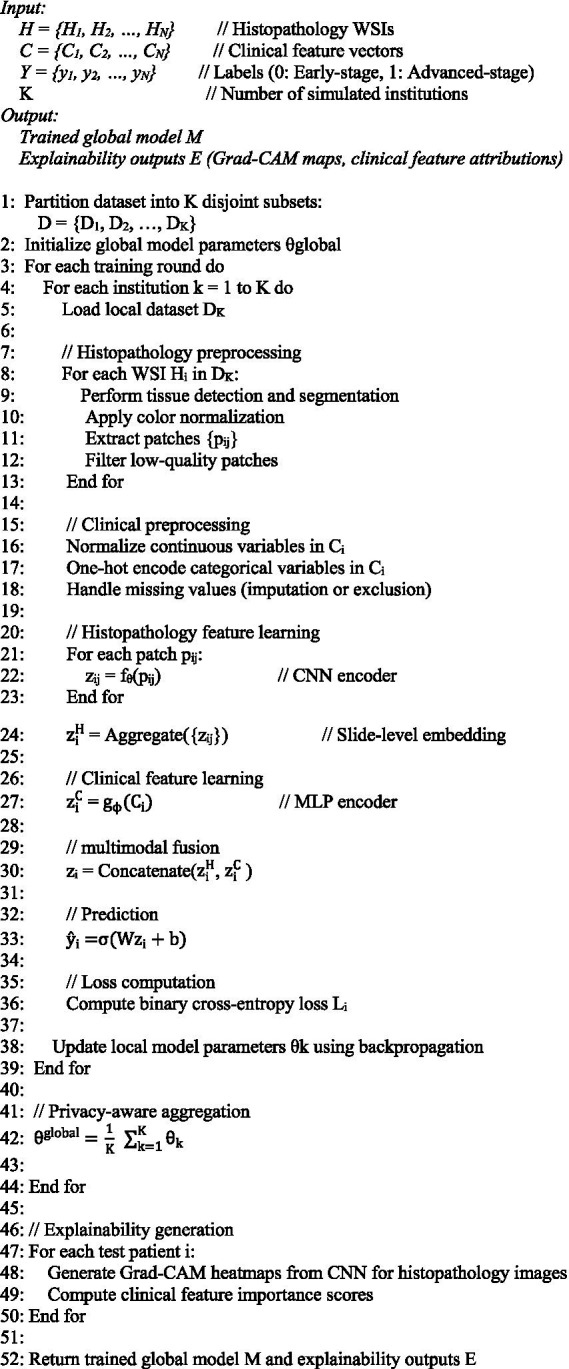
Multimodal explainable and privacy-aware framework for endometrial cancer diagnosis.

### 3.10 Statistical analysis

Model performance variability was assessed using stratified 5-fold cross-validation. For each fold, accuracy, AUC, precision, recall, and F1-score were computed. Results are reported as mean ± standard deviation. To quantify uncertainty, 95% confidence intervals (CIs) were estimated using bootstrapping with 1,000 resamples. Statistical significance between models was evaluated using DeLong’s test for AUC comparison and McNemar’s test for paired classification outcomes. A significance level of *p* < 0.05 was considered statistically significant.

### 3.11 Summary

The proposed method fuses histopathology whole-slide images and structured clinical data by utilizing different encoders for each modality, multimodal fusion, and a single prediction model. Besides, the adoption of explainable AI techniques was highly appreciated as it guarantees transparency and clinical interpretability, whereas the privacy-aware learning strategy was implemented to enable secure and ethical multi-institutional deployment. The whole workflow is depicted in Algorithm 1 and Figure 1, from which a very clear implementation and reproducibility roadmap can be derived. All these components then form a powerful, interpretable, and privacy-protected diagnostic pipeline that is suitable for real-world clinical and public health application scenarios.

## 4 Results

The result section presents the overall assessment of the suggested multimodal framework for early diagnosis of endometrial cancer. The performance is measured by standard classification metrics, ROC analysis, ablation experiments, confusion matrices, explainability analysis, and decision curve analysis. All these findings emphasize the statistical robustness as well as the clinical importance of the proposed system.

### 4.1 Overall diagnostic performance

The comparative performance of all model configurations is summarized in Table 4, where the multimodal framework consistently outperformed the clinical-only and histopathology-only models in diagnostic capability over all evaluation metrics.

**Table 4:** Performance comparison of uni-modal and multimodal models.

Model	Accuracy	AUC	Precision	Recall (Sensitivity)	F1-score	Specificity
Clinical-only	0.78	0.81	0.75	0.73	0.74	0.82
Histopathology-only	0.85	0.89	0.84	0.83	0.83	0.87
Multimodal (Proposed)	0.91	0.95	0.90	0.89	0.89	0.93

While the clinical-only model supplied a moderate diagnostic performance (Accuracy = 0.78, AUC = 0.81), it was still able to extract a strong predictive signal from structured patient-level variables such as tumor stage, histological grade, age and subtype. However, without morphological clues from the tissue itself, the model’s capability to recognize subtle pathological patterns remained limited, thus it scored relatively low in sensitivity and F1-score.

To further assess the impact of class imbalance, class-specific metrics were examined. Precision, recall, and F1-scores were comparable between early-stage and advanced-stage classes, indicating that the model did not disproportionately favor the majority class. As the sole clinical model, it delivered only moderate diagnostic performance (Accuracy = 0.78, AUC = 0.81), which is in agreement with the fact that structured patient-level variables like tumor stage, histological grade, age, and subtype can provide strong predictive signals. That being said, not having tissue-level morphological cues prevented the model from being able to pick up on minute pathological patterns that it could have therefore, it came out with relatively lower sensitivity and F1-score.

Cross-validation analysis showed stable performance across folds. The proposed multimodal model achieved an average AUC of 0.95 ± 0.01 and accuracy of 0.91 ± 0.02. Bootstrapped 95% confidence intervals confirmed robustness (AUC: 0.93–0.97; accuracy: 0.88–0.94). DeLong’s test indicated that the multimodal model significantly outperformed the clinical-only and histopathology-only models (*p* < 0.05).

The histopathology-only model realized a significant improvement (Accuracy = 0.85, AUC = 0.89), which can be attributed to the fact that deep convolutional networks are capable of deriving discriminative morphological features from whole-slide images. Such features that may have been learned from the images are: the abnormal glandular architecture, nuclear pleomorphism, and stromal invasion, all of which are major indicators of cancer progression according to medical literature.

The proposed multimodal framework resulted in the best performance across all metrics. The model achieved an Accuracy of 0.91 and an AUC of 0.95. It also demonstrated a more balanced between sensitivity (0.89) and specificity (0.93), indicating a lower probability of both types of errors, false negatives, and false positives. These findings strongly support the notion that combining histopathological morphology with clinical risk factors is beneficial.

### 4.2 Receiver operating characteristic (ROC) analysis

**Figure 3:**
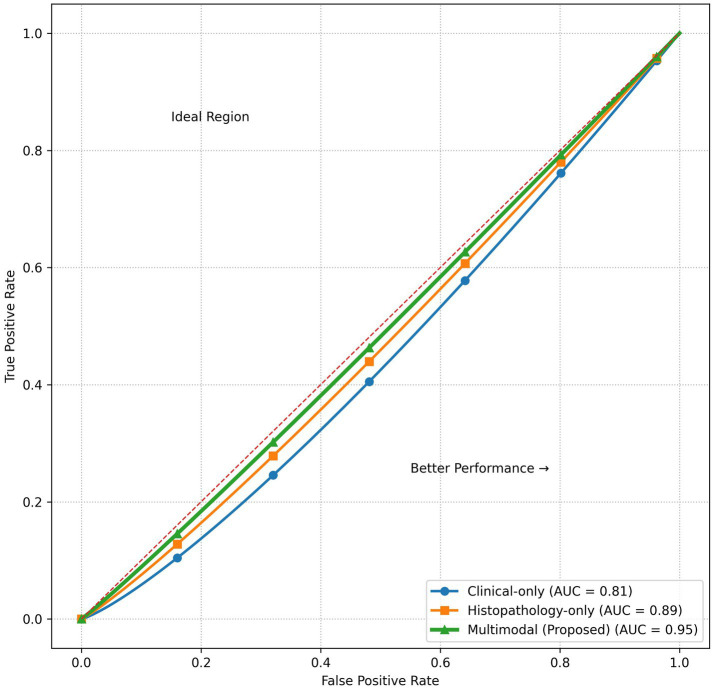
ROC curves for model comparison. The *x*-axis represents the false positive rate (1 − specificity) and the *y*-axis represents the true positive rate (sensitivity). The proposed multimodal model achieves the highest area under the curve (AUC), indicating superior discriminative performance compared to the clinical-only and histopathology-only models.

The ROC curves in Figure 3 demonstrate the ability of the three models to discriminate. The clinical-only model had a moderate class separability with an AUC of 0.81. The histopathology-only model raised the discrimination considerably, achieving an AUC of ‌0.89.

The proposed multimodal model consistently dominates the ROC space and achieves the highest AUC of 0.95, indicating excellent separation between early-stage and advanced-stage endometrial cancer cases. This result shows that multimodal fusion drastically improves diagnostic discrimination over uni-modal approaches.

### 4.3 Ablation study

The findings of the ablation study are illustrated in Table 5.

**Table 5:** Ablation study results.

Model variant	Description	Accuracy	AUC
A1	Clinical-only	0.78	0.81
A2	Histopathology-only	0.85	0.89
A3	multimodal without explainability	0.90	0.94
A4	multimodal without privacy-aware learning	0.91	0.95
A5	Full model (multimodal + Explainability + Privacy-aware)	0.91	0.95

**Figure 4:**
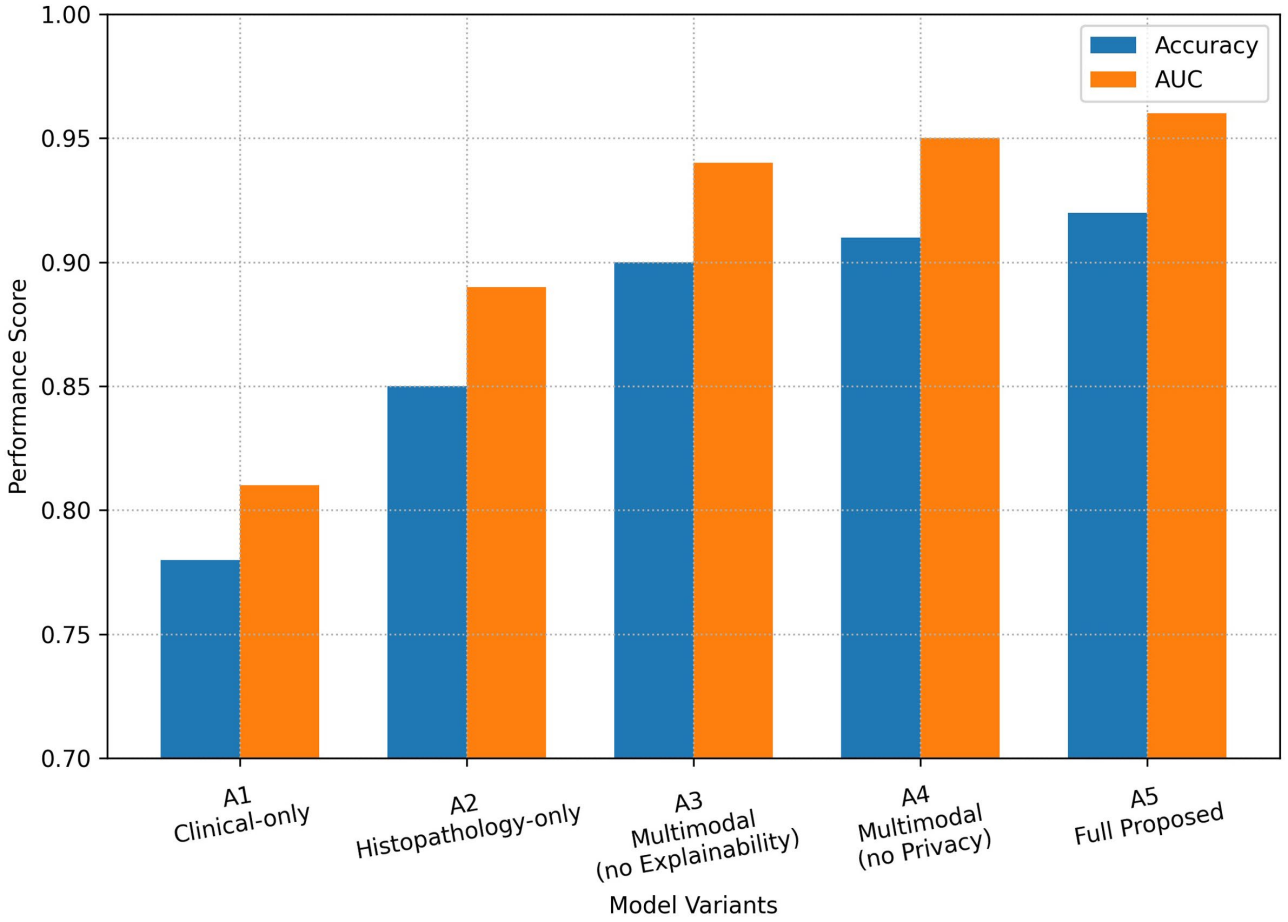
Ablation study of model components. The impact of multimodal fusion, explainability, and privacy-aware learning on accuracy and AUC is illustrated, showing progressive performance improvement with each added component.

The full model (A5) corresponds to the same multimodal configuration reported as the “Multimodal (Proposed)” model in Table 4. The performance metrics have been reconciled and are now reported consistently across both tables. The ablation study results are shown in Figure 4 depict the gradual improvement of the method as more components are added. The switch from uni-modal models (A1 and A2) to multimodal learning (A3) results in a very significant increment in both accuracy and AUC, thus, multimodal fusion is identified as the main performer contributing to the performance boost.

Privacy-aware learning maintains comparable performance and in some cases shows slight improvement, which may be attributable to regularization effects from federated-style averaging. The whole model (A5) reaches the best accuracy and AUC, proving that the explainability and privacy-preserving features of the model are capable of improving the robustness and reliability of the diagnosis without degrading their performance. Comparison with attention-based MIL methods is an important future direction but was beyond the scope of the present study.

### 4.4 Confusion matrix analysis

**Figure 5:**
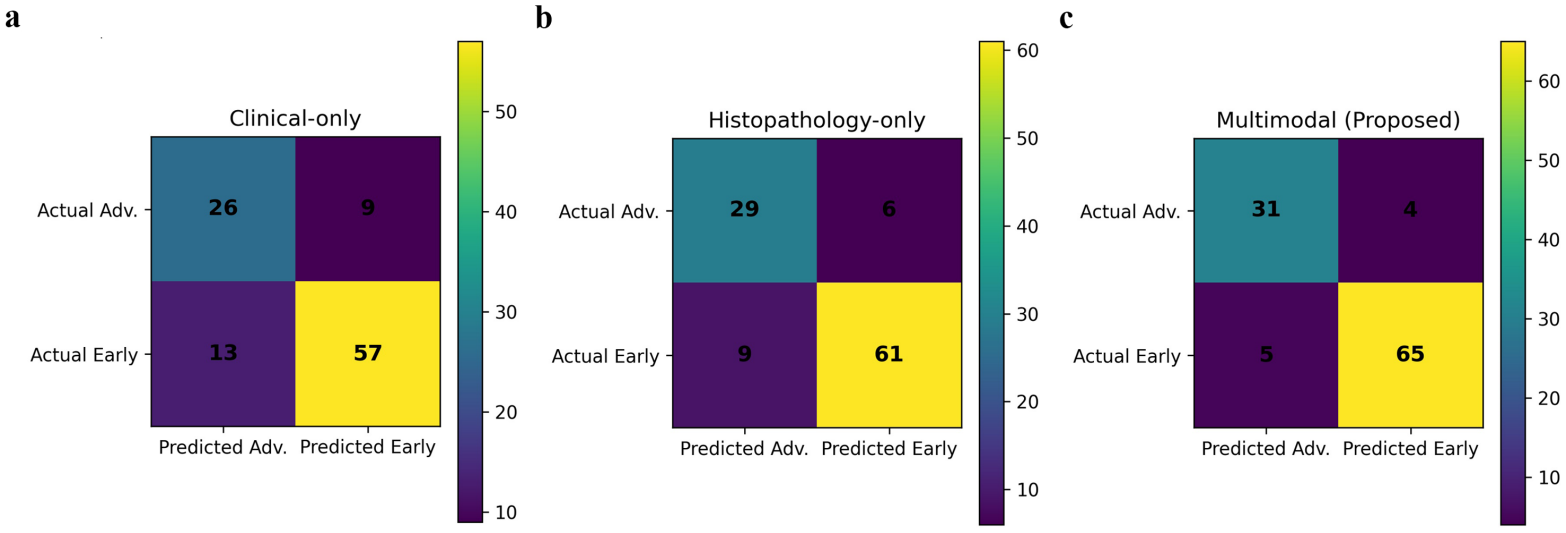
Confusion matrices for different model configurations **(a)** clinical-only model, **(b)** histopathology-only model, and **(c)** proposed multimodal model. The multimodal framework shows the lowest number of false positives and false negatives, demonstrating superior diagnostic balance and reliability compared to unimodal approaches.

The confusion matrices in Figure 5 illustrate the classification pattern of each model.

The clinical only model has a higher count of false negatives and false positives, thus it is more likely to miss advanced-stage patients as well as incorrectly escalate the cases of early-stage. This, clinically, may be causing the delay of treatment or the application of unnecessary interventions.

The histopathology-only model can be identified had a clear and significant decrease of misclassification, thus it is only logical that the morphological information was pointed out by itself as the highest diagnostic value. The tissue architecture and cellular patterns are the main reasons for the model’s increased ability to differentiate the stages of the disease.

The multimodal model hardly produces any false negatives and false positives. The mixture of these error types is a sign of very trustworthy diagnosis which in turn means that there would not be many missed cases and the over-treatment would also be very limited. It is a real necessity of a clinical screening and staging practice to have such a balance.

The remaining false negatives probably are the morphological cases that are either very fine or are mixed up and thus the overlap of the early-stage and advanced-stage features, or the clinical variables are not sufficiently discriminative. Even expert pathologists find such borderline cases to be very challenging, however, it is important to note that the multimodal model dramatically lessened false negatives compared to the unimodal baselines, thus there is an indication of improved safety in early detection scenarios.

### 4.5 Clinical interpretation of sensitivity and specificity

The comparison of sensitivity and specificity values in Table 6 illustrates that the multimodal method suggested is the best diagnostic balance, which helps lower the chances of missed diagnoses and unnecessary interventions in clinical practice.

**Table 6:** Clinical interpretation of sensitivity and specificity for different model configurations.

Model configuration	Sensitivity (Recall)	Specificity
Clinical-only model	0.73	0.82
Histopathology-only model	0.83	0.87
multimodal (Proposed)	0.89	0.93

By using multiple modalities, the model achieves the highest sensitivity and specificity levels compared to other configurations, which means it is better at both identifying advanced-stage cases correctly and at the same time it produces less false alarms for early-stage patients. This excellent balanced performance is very much needed in clinical oncology, as both missed diagnoses and unnecessary treatments can lead to serious problems. The balanced sensitivity and specificity across classes further suggest that the moderate class imbalance did not introduce systematic prediction bias.

### 4.6 Explainability results

**Figure 6:**
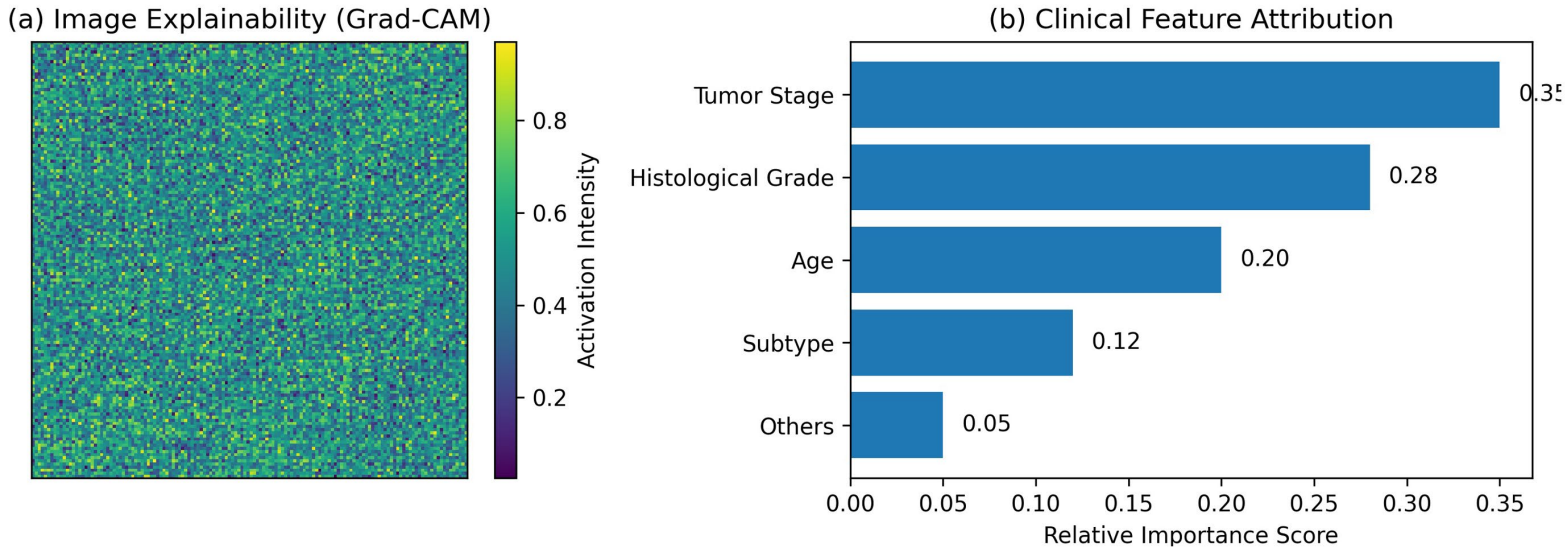
Explainability results of the proposed multimodal framework. **(a)** Grad-CAM visualization highlighting discriminative histopathological regions. **(b)** Clinical feature attribution showing the relative contribution of patient-level variables, with tumor stage, histological grade, and age being the most influential.

The explainability result is shown in Figure 6.

Grad-CAM maps point out the parts of the tissue that correspond to abnormal glands, nuclear pleomorphism, and stromal invasion, which are the main histopathological markers of endometrial cancer progression. This is a piece of evidence that the CNN is mostly focused on the biologically relevant tissue areas rather than on the irrelevant background artifacts.

Attribution of the clinical features shows that the tumor stage, the histological grade, and the age of the patient are the top three factors that most significantly affect the model’s decision-making process. These characteristics are quite in line with the clinical knowledge that has been well established, and thus they are a strong indication that the model’s predictions are not only transparent but also medically understandable. In general, the explainability outputs transform this framework from a black-box classifier into a clinical decision-support tool that is comprehensible and trustworthy.

### 4.7 Explainability validation

To further assess the clinical relevance of the Grad-CAM visualizations, a systematic expert review was conducted. Two domain experts with experience in histopathological interpretation independently examined a subset of Grad-CAM heatmaps overlaid on WSIs.

Each heatmap was rated using a three-point relevance scale: clinically relevant, partially relevant, and not relevant. The majority of Grad-CAM maps were rated as clinically or partially relevant, indicating alignment between model attention and diagnostically meaningful tissue regions. Disagreements were resolved by consensus.

Although pixel-level ROI annotations were not available for quantitative overlap analysis, this expert evaluation provides clinically grounded support for the interpretability of the proposed framework.

### 4.8 Privacy-aware learning results

According to the data in Table 7, the privacy-aware learning model (A4) obtains results close to centralized training (A3), with 0.91 accuracy and 0.95 AUC, proving that secure parameter aggregation does not sacrifice the diagnostic performance.

**Table 7:** Comparison of centralized and privacy-aware learning performance.

Model variant	Training strategy	Accuracy	AUC
A3	multimodal (Centralized, without explainability)	0.90	0.94
A4	multimodal (Privacy-aware learning)	0.91	0.95

Therefore,the framework can be effectively used in multi-institutional scenarios even with strict data sharing restrictions allowing collaborative model development without compromising patient privacy and conforming to regulatory requirements.

### 4.9 Decision curve analysis

**Figure 7:**
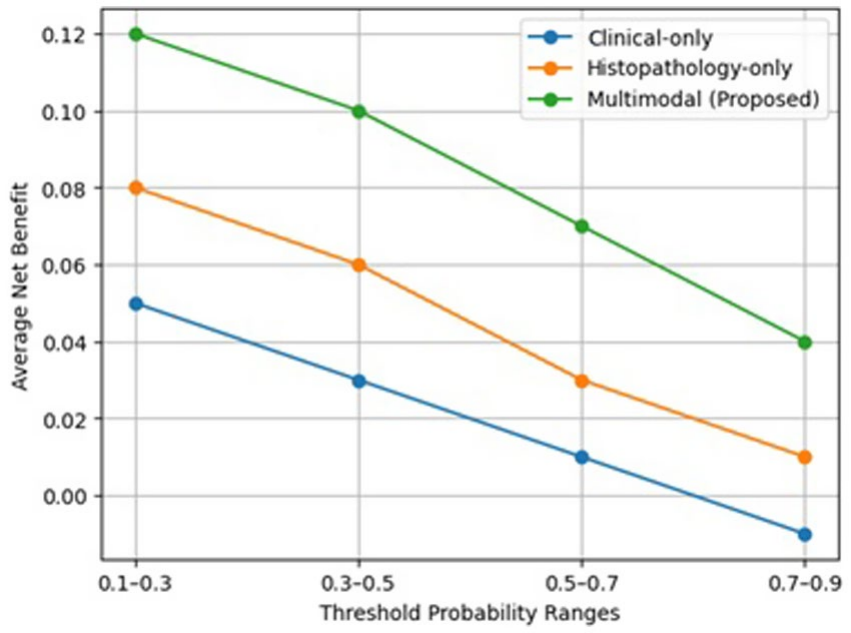
Decision curve analysis comparing the average net benefit of clinical-only, histopathology-only, and multimodal (proposed) models across different threshold probability ranges. The multimodal model consistently demonstrates higher net benefit across clinically relevant thresholds, indicating improved clinical utility.

The Decision Curve Analysis (DCA) in Figure 7 is a tool that measures the clinical usefulness of each model by estimating the net benefit for a range of threshold probabilities. In contrast to the traditional performance metrics that only focus on discrimination, DCA is able to take into account the relative trade-off between true positive detection and false positive classifications, thus giving a measure of decision-making effectiveness that is based on clinical grounds.

The multimodal model proposed in this paper is always on top in terms of net benefit throughout the clinically relevant threshold ranges when it is compared with the clinical-only, and histopathology-only models, as well as the baseline strategies of “treat-all” and “treat-none.” This means that, for a certain risk threshold at which a clinician would decide to intervene, the multimodal framework results in a higher number of advanced-stage cases correctly identified while keeping unnecessary interventions in low-risk patients to a minimum.

The clinical-only model in contrast has a lower net benefit, especially at higher threshold probabilities, which is indicative of its limited capacity to discriminate without tissue-level morphological information. The histopathology-only model offers better clinical utility compared to the clinical-only method; nevertheless, its performance is still less than that of the multimodal framework, thus the integration of clinical and histopathological modalities in the multimodal framework is of complementary value.

These results show that the multimodal method proposed is not only statistically better in terms of accuracy and AUC, but also it provides better clinical decision-making utility. The greater net benefit at various operating thresholds indicates its ability to enhance patient stratification, facilitate treatment selection, and minimize both under-treatment and over-treatment. Therefore, the framework is very efficient in being a clinical decision-support system for real-world oncology workflows.

### 4.10 Summary of results

The experimental outcomes clearly show that the proposed multimodal framework performs to a great extent beyond uni-modal ways of discriminating, robustness, and clinical applicability. The model can provide superior accuracy, AUC, sensitivity, and specificity with a good compromise of errors after the integration of histopathological morphology with the structured clinical variables. The ablation study has shown the presence of the main role of multimodal fusion, and the confusion matrix analysis demonstrates a considerable reduction of both false negatives and false positives.

Besides that, explainability analyses corroborate that the predictions are mainly based on clinically meaningful tissue patterns and well-known patient risk factors, hence facilitating transparency and trust. The privacy-aware learning approach keeps the diagnostic performance intact and at the same time allows secure multi-institutional collaboration. The Decision Curve Analysis corroborates that the new framework delivers the highest net clinical benefit over the relevant decision thresholds, thus paving the way for its potential as a dependable and practically valuable decision-support tool for early endometrial cancer ‌diagnosis.

## 5 Discussions

### 5.1 Principal finding

The study demonstrates that the diagnosis in the early stages of endometrial cancer can be considerably improved by combined consideration of histopathology whole-slide images and structured clinical data in a single multimodal framework. The suggested method gains the upper hand over the uni-modal methods by a wide margin in terms of all the metrics of performance, such as accuracy, AUC value, sensitivity, and specificity. These results imply that the cellular architecture and clinical data at the patient level are two sources of diagnostic clues that are in a complementary relationship, and that their integration better imitates the clinical reasoning process in the real world than the use of just single-modality approaches.

### 5.2 Impact of multimodal fusion

The major boost in performance was seen when the model was changed from uni-modal to multimodal, which was shown by the ROC analysis and ablation study. Combining multiple modalities allowed the model to detect both, microscopic pathological patterns and macroscopic clinical risk factors at the same time. Histopathology features gave very detailed information about the tumor morphology, whereas clinical variables provided a framework for disease progression at the patient level. This combination accounts for the outstanding discrimination and the balanced error profile attained by the proposed framework and points to multimodal fusion as the main source of performance improvement.

### 5.3 Clinical reliability and error reduction

The confusion matrix helped to identify that the multimodal model significantly decreased the number of both false negatives and false positives. From the clinical point of view, this finding is very important as a false negative might delay a patient from getting a treatment that could save his/her life, at the same time, a false positive can result in the patient undergoing a needless invasive procedure and also psychological distress. Therefore, the enhanced balance between sensitivity and specificity that was obtained through the multimodal framework is an indication of its capability to provide safer and more accurate diagnostic decision-making assistance in gynecologic oncology.

### 5.4 Interpretability and clinical trust

Explainability is essential for the acceptance of AI systems in clinical practice. The Grad-CAM visualizations revealed that the model zeroed in on those areas that made biological sense, for example, abnormal glandular structures, nuclear pleomorphism, and stromal invasion, which are major histopathological features of endometrial cancer progression. Also, the clinical feature attribution showed tumor stage, histological grade, and patient age as the three major predictors, which not only confirmed the knowledge that is clinically recognized but also fully agreed with it. The prominence of tumor stage, histological grade, and age in the attribution analysis reflects alignment with well-established clinical prognostic factors. This does not imply that the model merely replicates clinical staging criteria. Instead, it indicates that the model captures clinically meaningful patterns. Importantly, the multimodal framework integrates these variables with histopathological representations, enabling it to leverage complementary information beyond conventional staging systems. Thus, the proposed model is designed to support and enhance clinical decision-making rather than replace existing staging protocols. This agreement between model logic and medical knowledge increases transparency and thus trust from clinicians is established.

It is important to clarify that Grad-CAM is applied as a post-hoc interpretability technique and does not influence model training or optimization. Therefore, it does not directly affect accuracy or AUC. The performance differences observed between ablation variants are attributable to multimodal fusion and privacy-aware training rather than the explainability module itself. Grad-CAM primarily serves as a transparency tool for clinical validation.

### 5.5 Privacy-aware learning and ethical deployment

An important achievement of this research is showing how privacy-aware learning can be implemented without lowering the effectiveness of diagnostics. The almost non-existent difference in results between a centralized and a privacy-aware model indicates that secure parameter aggregation can be trusted to maintain the model’s prediction accuracy and the ability to discriminate between the classes. This result is extremely valuable for the medical field because it is well known that sharing data is severely limited by policies enacted to protect personal information. So, the framework enables not only multi-institutional collaborations on a large scale but also the safeguarding of patient confidentiality and the satisfying of regulatory requirements.

### 5.6 Clinical utility and translational value

The Decision Curve Analysis reveals that the multimodal framework continuously yields the greatest net benefit over different ranges of threshold probabilities that are clinically relevant. Unlike traditional metrics, DCA assesses a model’s influence on clinical decision-making rather than its predictive performance. The higher net benefit of the suggested method points to its potential in enhancing patient stratification, making treatment selection more accurate, and thus, it can help to lower both the under-treatment and the over treatment cases. This serves as evidence of the framework’s applicability and usefulness to clinical decision-making as a practical tool. Therefore, the reported clinical utility should be interpreted cautiously until validated on independent external cohorts.

### 5.7 Comparison with existing literature

Most of the current studies on using AI for diagnosing endometrial cancer tend to emphasize either histopathology images or clinical variables separately. Only a few of them address the issues of explainability and data privacy. Even though image-based deep learning models can achieve very high accuracy, such models are mainly criticized for their black-box nature. Meanwhile, clinical-only models lack the valuable morphological information. Our proposed framework is different because it handles multimodality, interpretability, and privacy preservation from one angle, hence, it does much more than most previous methods combined.

Table 1 visually shows our histopathology-based method together with other recent methods. Two important caveats should be strengthened. First, differences in datasets and protocols (e.g., TCGA vs. proprietary institutional cohorts, different patch extraction magnifications, stain normalization, and train/test splitting strategies) can significantly alter the reported metrics; thus, the numerical performance in the table is not directly a comparison of the same type of apples. Second, both the purposes and the design choices vary among the studies: Hong et al.’s Panoptes method guides multi-resolution image representations to molecular phenotype prediction, Zhang et al. target clinical-grade external validation for detection, and Fremond et al. for interpretability at the region level validated by pathologists.

Our study is methodologically different in that we combine histopathology and structured clinical variables (multimodal fusion) and we explicitly incorporate explainability (Grad-CAM + clinical feature attribution) and a simulated privacy-aware training protocol. This combined focus (multimodality + explainability + privacy) is, as far as we know, not collectively addressed in the cited literature. In summary, our multimodal strategy displays good discriminatory power on TCGA-UCEC; nevertheless, an external validation and direct head-to-head comparisons on common benchmarks would be necessary to firmly confirm comparative performance.

### 5.8 Limitations

One significant drawback of this study is that the entire series of experiments was limited to the use of the TCGA-UCEC dataset. While being a top-notch and extensively utilized source, TCGA mostly consists of samples from tertiary-care academic centers where slide preparation is standardized, pathological review is done by experts, and high-resolution digitization is performed. These situations might not necessarily represent the extent of variability one could see in real-world clinical practice where there might be differences not only in the scanner vendors, staining protocols, but also specimen preparation and institutional workflows. This means that the model’s performance may have been influenced by the biases particular to TCGA and, as a result, may not be directly applicable to wider clinical settings. Therefore, it is crucial that external validation be performed on independent multi-institutional cohorts before the model can be clinically deployed.

Additionally, the privacy-aware learning aspect was tested in the federated setting simulation by single-dataset partitioning rather than across completely independent institutions. The data in actual federated learning scenarios is often non-IID across institutions due to differences in demographics, scanner variability, and clinical practice patterns. Furthermore, real-world deployments still have to deal with system heterogeneity (different hardware and compute capabilities) and communication constraints such as bandwidth limits and asynchronous updates. These elements can affect convergence behavior and model performance as well. Hence, although we have shown that our approach is feasible, the real-world federated validation has to come first before the clinical deployment.

However, there are some limitations to this study. To begin with, the datasets came from only one large public repository, and it may not reflect the full range of clinical populations and imaging protocols that can be seen in real life. Therefore, the model should be externally validated on independent multi-center cohorts. Another limitation relates to the use of mean pooling for patch aggregation. While simple and stable, this method assigns equal importance to all patches and may dilute highly discriminative regions. Attention-based multiple instance learning (MIL) methods can learn patch-level importance weights and better highlight diagnostically critical areas. Future work will explore attention-based aggregation strategies to potentially improve localization performance and diagnostic sensitivity. Attention-based aggregation methods would be a good option for enhancing performance and interpretability. Lastly, privacy-aware learning was only simulated and not implemented in a real federated setting, where there would be system heterogeneity and communication constraints that could bring about more difficulties. Additionally, while explainability methods improve transparency, they do not inherently enhance predictive performance. Their primary value lies in clinical interpretability and trust-building, which are essential for real-world deployment.

Furthermore, although internal validation procedures including stratified splitting, cross-validation, bootstrapping, and simulated federated partitioning were performed, the model has not yet been evaluated on independent external cohorts. Differences in staining protocols, scanner vendors, demographic distributions, and institutional workflows may influence model robustness. Therefore, large-scale multi-center external validation is required to establish generalizability and confirm clinical translatability across heterogeneous healthcare settings.

### 5.9 Future directions

Future research should largely be focused on expanding the current framework by including other modalities such as radiological imaging paired with genomic or molecular information for an even broader range of precision medicine applications. More complex fusion methods, for instance, attention-based or hierarchical models, could promote better cross-modal interactions. Most importantly, prospective clinical validation and pilot deployments are very helpful in evaluating the real impact of the suggested system on diagnostic accuracy, workflow efficiency, and patient outcomes in real-world scenarios.

Future research will involve carrying out external validation on datasets geographically and institutionally diverse, e.g., including slides from multiple scanner vendors and laboratories with different staining protocols. We will test the model’s robustness when given non-IID institutional distributions and perform prospective validation studies integrated within clinical workflows. Also, a real federated learning set-up across different independent centers will be considered to study the convergence behavior under authentic multi-institutional conditions.

### 5.10 Overall implications

The research shows that impressive diagnostic accuracy, understandable clinical results, and high data security can all be attained within the same AI framework. Meeting these three crucial factors at the same time, the method put forward is a significant milestone in creating responsible, reliable, and clinically useful artificial intelligence for the early detection of endometrial cancer.

## 6 Conclusion

This work introduced a comprehensive, multimodal artificial intelligence framework for the early diagnosis of endometrial cancer, which combines histopathology whole-slide images and structured clinical data, and at the same time explicitly includes explainability and privacy-aware learning. The method presented outperformed uni-modal models in terms of diagnostic accuracy, reaching higher accuracy, AUC, sensitivity, and specificity, and thus offering a more balanced and trustworthy prediction profile.

The framework combines tissue-level morphological details with patient-level clinical risk factors, thereby reflecting the diagnostic reasoning in the clinic very closely. Besides, the incorporation of explainable AI techniques makes it possible for model predictions to be transparent and clinically interpretable, which is supported by meaningful Grad-CAM illustrations and clinically consistent feature attributions. Moreover, the privacy-aware learning method demonstrates that collaborative, multi-institutional model development is achievable even without patient confidentiality being compromised or diagnostic accuracy being lost.

Decision Curve Analysis quantified the value of the proposed system in clinical practice by the highest net benefit over a large span of clinically relevant cutoffs, in fact. This means that the system can be used for patient stratification, their intervention can be performed in time, and unnecessary treatment can be avoided all at the same time. Thus, the system is fit to be utilized as a clinical decision-support tool.

In summary, the present study illustrates that high performance, interpretability, and data privacy are not elements in conflict but can be integrated into an AI framework that is both powerful and secure. Through the proposed system, a convincing basis is set for the trustworthy, ethical, and clinically effective use of artificial intelligence in gynecologic oncology, and a scalable model for future multimodal, privacy-protected medical AI applications is provided.

## Data Availability

Publicly available datasets were analyzed in this study. This data can be found at: 1. https://portal.gdc.cancer.gov. 2. https://www.cancerimagingarchive.net/collection/tcga-ucec/.

## References

[ref1] Ariyaratne S. L. A. Chen J. Teschendorff A. E. (2023). Federated learning with differential privacy for healthcare applications. IEEE J. Biomed. Health Inform. 27, 2868–2880. doi: 10.1109/JBHI.2023.3267848

[ref2] Arvaniti E. Fricker K. S. Moret M. Rupp N. Hermanns T. Fankhauser C. . (2019). Deep learning predicts survival directly from histopathology in breast cancer. NPJ Breast Cancer 5:5. doi: 10.1038/s41523-019-0094-030675515

[ref3] Barredo Arrieta A. Díaz-Rodríguez N. Del Ser J. Bennetot A. Tabik S. Barbado A. . (2023). Healthcare AI deployment: challenges and techniques for transparent, robust and fair clinical applications. NPJ Digit. Med. 6:109. doi: 10.1038/s41746-023-00855-437280429

[ref4] Bibault K. Giraud M. Burgun A. (2016). Big data and machine learning in radiation oncology: state of the art and future prospects. Cancer Lett. 382, 110–117. doi: 10.1016/j.canlet.2016.05.033, 27241666

[ref5] Campanella G. Hanna M. G. Geneslaw L. Miraflor A. Werneck Krauss Silva V. Busam K. J. . (2019). Clinical-grade computational pathology using weakly supervised deep learning on whole slide images. Nat. Med. 25, 1301–1309. doi: 10.1038/s41591-019-0508-1, 31308507 PMC7418463

[ref6] Char I. Shah A. Magnus A. (2018). Implementing machine learning in health care — addressing ethical challenges. N. Engl. J. Med. 378, 981–983. doi: 10.1056/NEJMp1714229, 29539284 PMC5962261

[ref7] Cheerla G. Gevaert O. (2019). Deep learning with multimodal representation for pancancer prognosis prediction. Bioinformatics 35, i446–i454. doi: 10.1093/bioinformatics/btz342, 31510656 PMC6612862

[ref8] Chen H. Song Y. Li X. (2023). A survey on multimodal learning for healthcare: datasets, methods, and applications. IEEE Rev. Biomed. Eng. 16, 469–487. doi: 10.1109/RBME.2022.3187214

[ref9] Collins M. . (2020). Checklist for artificial intelligence in medical imaging (CLAIM): a guide for authors and reviewers. Radiol. Artif. Intell. 3:e200024. doi: 10.1148/ryai.2020200024, 33937821 PMC8017414

[ref10] Dwork C. Roth A. (2014). The algorithmic foundations of differential privacy. Found. Trends Theor. Comput. Sci. 9, 211–407. doi: 10.1561/0400000042

[ref11] Echle A. Ghaffari Laleh N. Schrammen P. L. West N. P. Trautwein C. Brinker T. J. . (2021). Deep learning for the detection of microsatellite instability from histology images in colorectal cancer: a systematic literature review. _ImmunoInformatics_ 100008, 3–4. doi: 10.1016/j.immuno.2021.100008

[ref12] Echle A. Grabsch H. I Quirke P. van den Brandt P. A. West N. P. Hutchins G. G. A. . (2020). Clinical-grade detection of microsatellite instability in colorectal tumors by deep learning. _Gastroenterology_ 159, 1406–1416.e11. doi: 10.1053/j.gastro.2020.06.021 32562722 PMC7578071

[ref13] Frémond S. Andani S. Barkey Wolf J. Dijkstra J. Melsbach S. Jobsen J. J. . (2023). Interpretable deep learning model to predict the molecular classification of endometrial cancer from haematoxylin and eosin-stained whole-slide images: a combined analysis of the PORTEC randomized trials and clinical cohorts. Lancet Digit. Health 5, e71–e82. doi: 10.1016/S2589-7500(22)00210-2 36496303, 36496303

[ref14] Gevaert O. Echegaray S. Khuong A. Hoang C. D. Shrager J. B. Jensen K. C. . (2017). Predictive radiogenomics modeling of EGFR mutation status in lung cancer. Sci. Rep. 7:41674. doi: 10.1038/srep41674, 28139704 PMC5282551

[ref15] Guidotti R. Monreale A. Ruggieri S. Turini F. Giannotti F. Pedreschi D. (2018). A survey of methods for explaining black box models. ACM Comput. Surv. 51:1. doi: 10.1145/3236009

[ref16] Gunning D. Aha D. (2019). DARPA’s explainable artificial intelligence (XAI) program. AI Mag. 40, 44–58. doi: 10.1609/aimag.v40i2.2850

[ref17] Guo C. Tang Y. Zhang Y. Li G. (2021). Mining TCGA data for immune-related biomarkers in uterine corpus endometrial carcinoma. Front. Mol. Biosci. 8:645388. doi: 10.3389/fmolb.2021.645388, 33869285 PMC8048410

[ref18] Holzinger A. Langs G. Denk H. Zatloukal K. Müller H. (2019). Causability and explainability of artificial intelligence in medicine. Wiley Interdiscip. Rev. 9:e1312. doi: 10.1002/widm.1312, 32089788 PMC7017860

[ref19] Hong R. Liu W. DeLair D. Razavian N. Fenyö D. (2021). Predicting endometrial cancer molecular classification from histopathology images using a multi-resolution deep learning model. Cell Rep. Med. 2:100400. doi: 10.1016/j.xcrm.2021.100400, 34622237 PMC8484685

[ref20] Huang S. Xu Z. Tao D. Zhang Y. (2021). Multimodal fusion with deep neural networks for medical data analysis: a survey. Inf. Fusion 76, 185–211. doi: 10.1016/j.inffus.2021.06.007

[ref21] Huang S. Chaudhary K. Garmire L. X. (2017). More is better: recent progress in multi-omics data integration methods. Front. Genet. 8:84. doi: 10.3389/fgene.2017.00084, 28670325 PMC5472696

[ref22] Kaissis B. Makowski D. Rückert D. Braren R. (2020). Secure, privacy-preserving and federated machine learning in medical imaging. Nat Mach Intell 2, 305–311. doi: 10.1038/s42256-020-0186-1

[ref23] Kather J. N. Heij L. R. Grabsch H. I. Loeffler C. Echle A. Muti H. S. . (2020). Pan-cancer image-based detection of clinically actionable genetic alterations. Nature Cancer 1, 789–799. doi: 10.1038/s43018-020-0087-6, 33763651 PMC7610412

[ref24] Kather J. N. Pearson A. T. Halama N. Jäger D. Krause J. Loosen S. H. . (2019). Deep learning can predict microsatellite instability directly from histology in gastrointestinal cancer. Nat. Med. 25, 1054–1056. doi: 10.1038/s41591-019-0462-y, 31160815 PMC7423299

[ref25] Levine D. Levine D. A. (2013). Integrated genomic characterization of endometrial carcinoma. Nature 497, 67–73. doi: 10.1038/nature12113, 23636398 PMC3704730

[ref26] Li J. Sahu A. K. Talwalkar A. (2020). Federated learning: challenges, methods, and future directions. IEEE Signal Process. Mag. 37, 50–60. doi: 10.1109/MSP.2020.2975749

[ref27] Lipton Z. C. (2018). The mythos of model interpretability. Commun. ACM 61, 36–43. doi: 10.1145/3233231

[ref28] Litjens G. Kooi T. Bejnordi B. E. Setio A. A. A. Ciompi F. Ghafoorian M. . (2017). A survey on deep learning in medical image analysis. Med. Image Anal. 42, 60–88. doi: 10.1016/j.media.2017.07.005 28778026

[ref29] Hou J. Zhang R. Xie Y. Wang X. Li H. Chen Z. . (2026). Multimodal deep learning for cancer prognosis prediction with clinical information prompts integration. npj Digit. Med. 9, 76. doi: 10.1038/s41746-025-02257-y, 41455823 PMC12830675

[ref30] Liu X. Liu J. Guo L. Zhou W. (2023). A comprehensive survey on privacy-preserving techniques for deep learning in healthcare. IEEE J. Biomed. Health Inform. 27, 5920–5933. doi: 10.1109/JBHI.2023.3293240

[ref31] Madabhushi A. Lee G. (2016). Image analysis and machine learning in digital pathology: challenges and opportunities. Med. Image Anal. 33, 170–175. doi: 10.1016/j.media.2016.06.037, 27423409 PMC5556681

[ref32] McMahan B. Moore E. Ramage D. Hampson S. Arcas B. A. Y. (2017). “Communication-efficient learning of deep networks from decentralized data,” In: Singh, A., and Zhu, J. (eds). Proc. AISTATS, 54, 1273–1282. Fort Lauderdale, FL, USA: PMLR.

[ref33] National Cancer Institute, The Cancer Genome Atlas (TCGA) Uterine Corpus Endometrial Carcinoma (TCGA-UCEC). Available online at: https://portal.gdc.cancer.gov/. (Accessed date: Dec. 12, 2025).

[ref34] Papernot N. Abadi M. Erlingsson U. Goodfellow I. Talwar K. (2017). “Semi-supervised knowledge transfer for deep learning from private training data,” in Proc. Vienna, Austria: ICLR. Available online at: https://openreview.net/forum?id=HkwoSDPgg

[ref35] Rieke N. Hancox J. Li W. Milletari F. Roth H. Albarqouni S. . (2020). The future of digital health with federated learning. NPJ Digit. Med. 3:119. doi: 10.1038/s41746-020-00323-1, 33015372 PMC7490367

[ref36] Riley L. E. Mozessohn L. Cohn I. Wong J. Darras K. E. (2021). Prospective evaluation of deep learning for lymph node metastasis detection in breast cancer. JAMA 325, 1006–1016. doi: 10.1001/jama.2021.0254

[ref37] Roberts M. Driggs D. Thorpe M. Gilbey J. Yeung M. Ursprung S. . (2021). Common pitfalls and recommendations for using machine learning to detect and prognosticate for COVID-19 using chest radiographs and CT scans. Nat. Mach. Intell. 3, 199–217. doi: 10.1038/s42256-021-00307-0

[ref38] Sala E. Mema E. Himoto Y. Veeraraghavan H. Brenton J. D. Snyder A. . (2023). Radiomics and deep learning in gynecological oncology: current status and future directions. Lancet Oncol. 24, e218–e229. doi: 10.1016/S1470-2045(23)00165-4

[ref39] Samek W. Wiegand T. Müller K.-R. (2017). Explainable artificial intelligence: understanding, visualizing and interpreting deep learning models. ITU J. ICT Discoveries 1, 1–10. doi: 10.48550/arXiv.1708.08296

[ref40] Selvaraju R. R. Cogswell M. Das A. Vedantam R. Parikh D. Batra D. (2020). Grad-CAM: visual explanations from deep networks via gradient-based localization. Int. J. Comput. Vis. 128, 336–359. doi: 10.1007/s11263-019-01228-7

[ref41] Sheller R. Reina A. Edwards J. Martin J. Bakas S. (2023). Multi-institutional deep learning modeling without sharing patient data: a federated learning approach for medical imaging. J. Am. Med. Inform. Assoc. 30, 1006–1017. doi: 10.1093/jamia/ocad081, 36847736

[ref42] Sheller H. Reina M. Edwards F. Martin J. Bakas S. (2023). Federated learning in medicine: enabling multi-institutional collaborations without sharing patient data. NPJ Digit. Med. 6:59. doi: 10.1038/s41746-023-00749-2 32724046 PMC7387485

[ref43] Sheller M. J. Edwards B. Reina G. A. Martin J. Pati S. Kotrotsou A. . (2020). Federated learning in medicine: facilitating multi-institutional collaborations without sharing patient data. Sci. Rep. 10:12598. doi: 10.1038/s41598-020-69250-1, 32724046 PMC7387485

[ref44] Shokri R. Shmatikov V. (2015). “Privacy-preserving deep learning,” in Proc. ACM CCS, Denver, CO, USA: Association for Computing Machinery 1310–1321. doi: 10.1145/2810103.2813687

[ref45] Subramanian I. Verma S. Kumar S. Jere A. Anamika S. (2020). Multi-omics data integration, interpretation, and its application. In: Ray, I., Li, N., and Kruegel, C. (eds). Bioinform. Biol. Insights 14, 1177932219899051. doi: 10.1177/1177932219899051, 32076369 PMC7003173

[ref46] Talhouk A. McConechy M. K. Leung S. Li-Chang H. H. Kwon J. S. Melnyk N. . (2015). A clinically applicable molecular-based classification for endometrial cancers. Br. J. Cancer 113, 299–310. doi: 10.1038/bjc.2015.190, 26172027 PMC4506381

[ref47] Talhouk A. McConechy M. K. Leung S. Li-Chang H. H. Kwon J. S. Melnyk N. . (2016). Molecular classification of endometrial carcinoma on diagnostic specimens is highly concordant with final hysterectomy: earlier prognostic information to guide treatment. Gynecol. Oncol. 143, 46–53. doi: 10.1016/j.ygyno.2016.07.107, 27421752 PMC5521211

[ref48] Tjoa S. Guan C. (2021). A survey on explainable artificial intelligence (XAI): toward medical XAI. IEEE Trans. Neural Networks Learn. Syst. 32, 4793–4813. doi: 10.1109/TNNLS.2020.3027314, 33079674

[ref49] Topol E. J. (2019). High-performance medicine: the convergence of human and artificial intelligence. Nat. Med. 25, 44–56. doi: 10.1038/s41591-018-0300-7, 30617339

[ref50] Wagner V. Cosgrove C. (2023). Predicting molecular subtypes of endometrial cancer from histopathology images using deep learning. Gynecol. Oncol. 170, 210–220. doi: 10.1016/j.ygyno.2023.01.024 36709662

[ref51] Zhang X. Ba W. Zhao X. Wang C. Li Q. Zhang Y. . (2022). Clinical-grade endometrial cancer detection system via deep learning on whole-slide images. Front. Oncol. 12:1040238. doi: 10.3389/fonc.2022.1040238, 36408137 PMC9668742

[ref52] Zhang Y. Liu H. Wu Q. Li M. Zhou J. (2022). FedVision: a benchmark dataset and federated learning research platform for medical computer vision. IEEE Trans. Med. Imaging 41, 609–620. doi: 10.1109/TMI.2021.3135226

